# Evaluating the Therapeutic Efficacy of Mono- and Bivalent Affibody-Based Fusion Proteins Targeting HER3 in a Pancreatic Cancer Xenograft Model

**DOI:** 10.3390/pharmaceutics12060551

**Published:** 2020-06-13

**Authors:** Charles Dahlsson Leitao, Sara S. Rinne, Mohamed Altai, Olga Vorontsova, Finn Dunås, Per Jonasson, Vladimir Tolmachev, John Löfblom, Stefan Ståhl, Anna Orlova

**Affiliations:** 1 Department of Protein Science, School of Engineering Sciences in Chemistry, Biotechnology and Health, KTH Royal Institute of Technology, 106 91 Stockholm, Sweden; chdl@kth.se (C.D.L.); lofblom@kth.se (J.L.); 2 Department of Medicinal Chemistry, Uppsala University, 751 23 Uppsala, Sweden; sara.rinne@ilk.uu.se (S.S.R.); or mohamed.altai@med.lu.se (M.A.); 3 Department of Immunology, Genetics and Pathology, Uppsala University, 751 85 Uppsala, Sweden; olga.vorontsova@igp.uu.se (O.V.); vladimir.tolmachev@igp.uu.se (V.T.); 4 Division of Oncology and Pathology, Department of Clinical Sciences, Lund University, 221 00 Lund, Sweden; 5 Affibody AB, 171 65 Solna, Sweden; finn.dunas@affibody.se (F.D.); per.jonasson@affibody.se (P.J.); 6 Research Centrum for Oncotheranostics, Research School of Chemistry and Applied Biomedical Sciences, Tomsk Polytechnic University, 634050 Tomsk, Russia; 7 Science for Life Laboratory, Uppsala University, 751 23 Uppsala, Sweden

**Keywords:** affibody molecules, HER3, albumin-binding domain, seribantumab, therapy, MM-121

## Abstract

Human epidermal growth factor receptor 3 (HER3) has been increasingly scrutinized as a potential drug target since the elucidation of its role in mediating tumor growth and acquired therapy resistance. Affibody molecules are so-called scaffold proteins with favorable biophysical properties, such as a small size for improved tissue penetration and extravasation, thermal and chemical stability, and a high tolerance to modifications. Additionally, affibody molecules are efficiently produced in prokaryotic hosts or by chemical peptide synthesis. We have previously evaluated the biodistribution profiles of five mono- and bivalent anti-HER3 affibody molecules (designated as 3) fused to an albumin-binding domain (designated as A), 3A, 33A, 3A3, A33, and A3, that inhibit ligand-dependent phosphorylation. In the present study, we examined the therapeutic efficacy of the three most promising variants, 3A, 33A, and 3A3, in a direct comparison with the HER3-targeting monoclonal antibody seribantumab (MM-121) in a preclinical BxPC-3 pancreatic cancer model. Xenografted mice were treated with either an affibody construct or MM-121 and the tumor growth was compared to a vehicle group. Receptor occupancy was estimated by positron emission tomography/computed tomography (PET/CT) imaging using a HER3-targeting affibody imaging agent [^68^Ga]Ga-(HE)_3_-Z_08698_-NODAGA. The affibody molecules could inhibit ligand-dependent phosphorylation and cell proliferation in vitro and demonstrated tumor growth inhibition in vivo comparable to that of MM-121. PET/CT imaging showed full receptor occupancy for all tested drug candidates. Treatment with 3A and 3A3 affibody constructs was more efficient than with 33A and similar to the anti-HER3 antibody seribantumab, showing that the molecular design of affibody-based therapeutics targeting HER3 in terms of the relative position of functional domains and valency has an impact on therapeutic effect.

## 1. Introduction

New insights into tumor biology, gained from the perpetually growing field of oncology, have prompted researchers to contrive novel treatment strategies and regimens with the aim of reducing tumor burden and improving quality of life [1]. Prodigious work has been done to explore the antibody architecture, which demonstrates remarkable success in the clinic as therapeutic agents [2]. A plethora of antibody strategies is currently being investigated in clinical studies. Approaches include evaluating multiple specificities, valences, and combinations thereof for improved and more selective tumor targeting or for immune system co-engagement [3]. The conjugation of cytotoxic drugs has been widely used to increase potency [4], which has become even more relevant with strategies that increase the selectivity for tumor tissues, e.g., by using prodrug formats of antibodies [5,6].

In addition to monoclonal antibodies and antibody derivatives, several non-immunoglobulin scaffold affinity proteins have been developed [7]. One such scaffold is the affibody molecule, a small (approximately 7 kDa) three-helical bundle consisting of 58 amino acids based on the staphylococcal protein A derived Z-domain [8]. Target-specific affibody molecules are developed from combinatorial protein engineering by randomization of typically 13 surface exposed positions. The scaffold exhibits desirable biophysical properties with regards to small size for improved tissue penetration and extravasation, high stability, and fast refolding kinetics. Amenability to the modular extension of the molecule with additional functional domains provides a versatile framework for novel drug design. Some examples include the modulation of valency, specificity, linker lengths, and other functionalities, such as albumin-binding for a prolonged circulatory half-life. The scaffold is devoid of native cysteines, which enables straightforward cytoplasmic protein production in bacterial hosts and site-directed modifications by the introduction of terminal cysteines, including the incorporation of cytotoxic drugs for increased potency and chelators for labeling with radionuclides. Rapid renal clearance, as a consequence of its small size, makes the scaffold an excellent probe for companion diagnostics and other imaging applications [8].

Receptor tyrosine kinases in cancer have for decades been recognized as excellent targets for therapeutic intervention [9]. In particular, human epidermal growth factor receptors (HER) have been extensively scrutinized for their pivotal role in numerous malignancies. Perhaps the most studied of these receptors is the HER2, which is upregulated in 25–30% of breast cancer patients [10]. Potent HER signaling complexes are formed by the homo- or heterodimerization of the HER family members, which activate downstream signaling pathways that mediate cellular proliferation, migration, and survival [11]. The dysregulation of these receptors might convert otherwise innocuous cellular processes into transforming oncogenic signals. HER3 has attracted much attention in recent years for its important function as a driver of tumor progression and acquired treatment resistance [12]. The overexpression of this receptor, which occurs at a high frequency in several types of cancer, including breast, pancreatic, and ovarian cancer, has been associated with the decreased survival of patients, in particular with concomitant HER2 overexpression [13]. HER3 adopts a dimer-competent conformation upon the binding of its cognate ligands. Although the kinase activity of HER3 is impaired, the potent activation of downstream signaling pathways is achieved by heterodimerization with a kinase functional coreceptor, preferentially HER2, resulting in the transphosphorylation of six tyrosine residues situated on the cytosolic tail. The phosphotyrosines of HER3 act as docking sites for the initiation of signaling cascades, especially via the direct binding of the p85 subunit of PI-3K [14]. Furthermore, the HER3/HER2 heterodimer is thought to be the most potent mitogenic unit within the HER family and has a crucial function for HER2-mediated transformation and the continued growth of breast cancer cell lines driven by HER2 overexpression, demonstrated by two different HER3 knockdown models [15,16]. In clinical studies, it was demonstrated that a combination of anti-HER2 and anti-HER3 immunotherapies resulted in an encouraging overall response rate of 55% [17]. Thus, anti-HER3 therapies would be desirable for the treatment of patients with HER3-driven resistance.

There is currently no FDA-approved treatment specifically targeting HER3 and the two most prominent HER3-targeting antibody drug candidates, patritumab and seribantumab, were terminated in late-phase clinical trials for lack of efficacy (NCT02134015, NCT03241810). Nonetheless, numerous HER3-targeting strategies are being evaluated as a single or combination therapies in early stages of clinical development [12]. As it stands, HER3-mediated resistance to HER-targeted treatments, which encompass a wide range of therapeutics [18,19,20], poses major challenges for cancer therapy and calls for new and improved HER3-targeting therapeutics to be developed.

We have previously investigated the potential for affibody-based therapeutics targeting HER3 by evaluating the bivalent affibody-based construct TAM-HER3 in a preclinical in vivo model [21]. The molecule consists of two identical HER3-targeting affibody domains flanking an albumin-binding domain (ABD). The affibody domain Z_HER3:08698_ has previously been selected by our group and binds HER3 antagonistically in the picomolar range with cross-reactivity to the mouse ortholog [22]. The engineered 46-residue (approximately 5.2 kDa) ABD binds human serum albumin with femtomolar affinity and was included for the extension of construct half-life in circulation [23]. In order to elucidate the influence of domain orientation and valency on in vivo tumor targeting and biodistribution properties, five ABD-fused HER3-targeting variants were designed in either a mono- or bivalent format [24]. The monovalent (Z_HER3:08698_-G_3_S-ABD_035_, ABD_035_-G_3_S-Z_HER3:08698_), denoted by 3A and A3 respectively, and bivalent variants (Z_HER3:08698_-G_3_S-ABD_035_-G_3_S-Z_HER3:08698_, Z_HER3:08698_-G_3_S-Z_HER3:08698_-G_3_S-ABD_035_, ABD_035_-G_3_S-Z_HER3:08698_-G_3_S-Z_HER3:08698_), denoted by 3A3, 33A, and A33, respectively, could all inhibit heregulin-induced phosphorylation in HER3-expressing BxPC-3 and DU-145 cell lines [24]. Biodistribution studies conducted in mice bearing BxPC-3 xenografts revealed disparities in blood retention and organ uptake, most notably in excretory organs and in the tumors. Formats with an ABD located at the C-terminus (3A, 33A), as well as 3A3, provided the most favorable biodistribution profiles for tumor targeting in vivo. Additionally, differences in the internalization rate were observed for the studied variants in a panel of cell lines with varying levels of HER3 expression. The ability to induce rapid internalization is of relevance when designing payload delivery, which could be a viable strategy for HER3-targeted therapy [25]. Based on our results, we hypothesized that the molecular design of affibody-based therapeutics targeting HER3 in terms of the relative position of functional domains and valency has an impact on tumor targeting, biodistribution properties, and internalization rates.

The aim of the present study is to expand on previous findings that revealed a significant influence of molecular design on the in vivo targeting properties and pharmacokinetics of HER3-targeting affibody constructs [24]. Based on biodistribution data, the most promising formats (3A3, 33A, and 3A) were selected for the evaluation of therapeutic potential in a preclinical therapy efficacy study using a pancreatic adenocarcinoma xenograft model (BxPC-3). HER3 is often overexpressed in pancreatic cancer and is critical for mediating tumorigenesis via transactivation by epidermal growth factor receptor (EGFR) [26]. Both 3A3 and 3A demonstrated similar efficacy to MM-121 in terms of tumor growth inhibition and the prolonged survival of treated mice, whereas moderate efficacy was observed for 33A. No apparent side effects were observed for the affibody variants.

## 2. Materials and Methods

### 2.1. General

In vitro and in vivo studies were performed on BxPC-3 cells, primary pancreatic adenocarcinoma, obtained from the American Type Culture Collection (ATCC) via LGC Promochem, Borås, Sweden. Cells were cultured according to ATCC recommendations. In vivo experiments were planned and performed in accordance with national legislation on laboratory animal protection and were approved by the Ethics Committee for Animal Research in Uppsala, Sweden (animal permission C143/14, approved 16 September 2014).

Data were evaluated either by an unpaired, two-tailed *t*-test or by one-way ANOVA with Bonferroni correction for multiple comparisons using GraphPad Prism (version 6 for Windows GraphPad Software) in order to determine statistically significant differences (*p* < 0.05). Obtained values are presented as an average with standard deviation if not stated otherwise.

### 2.2. Production and HSA Purification

Genes for 3A3, 33A, and 3A, identical to previously investigated constructs [24] but lacking C-terminal cysteine, were subcloned into a pET45b(+) vector (Thermo Scientific, Chicago, IL, USA). The plasmids were transformed into BL21*(DE3) Escherichia coli (*E. coli*) (Thermo Scientific, Chicago, IL, USA) using a standard heat-shock protocol. Protein production was performed using fed-batch cultivation in a 6 × 1 L multifermenter system (GRETA4, Belach Bioteknik AB, Stockholm, Sweden). Shake flasks containing TSB + Y medium and 100 μg/mL carbenicillin were inoculated with single colonies and placed in a shake incubator at 37 °C and 180 rpm for 7 h. The inocula for each construct were added to separate fermenters and induced with a final concentration of 0.5 mM isopropyl β-d-1-thiogalactopyranoside (IPTG). Protein expression proceeded for 5 h before harvest. The harvested cells were lysed using a French press and the proteins were recovered with affinity chromatography on an ÄKTApure system (GE Healthcare, Uppsala, Sweden) using human serum albumin (HSA) immobilized on a Sepharose matrix. The column was equilibrated with TST buffer (25 mM Tris-HCl, 1 mM EDTA, 200 mM NaCl, 0.05% Tween, pH 8.0), which was used as a running buffer. The loaded sample was washed with ammonium acetate (5 mM, pH 5.5), followed by the isocratic elution of bound proteins with acetic acid (0.5 M, pH 2.8).

### 2.3. Reversed-Phase Chromatography

The eluate from the HSA purifications was adjusted to an acetonitrile (ACN) concentration of 10%, sterile filtered, and applied on a HR16/10 column (GE Healthcare, Uppsala, Sweden) packed with Source 15RPC (GE Healthcare, Uppsala, Sweden) previously equilibrated with RPC A-buffer (0.1% TFA, 10% ACN, 90% MQ water). RPC purification was performed on an ÄKTAexplore 100 (GE Healthcare, Uppsala, Sweden) with a flow rate of 6 mL/min. After sample loading, the column was washed with RPC A-buffer. After the column wash, bound protein was eluted with a linear gradient 0–50% RPC B-buffer (0.1% TFA, 80% ACN, 20% MQ water).

### 2.4. Buffer Exchange, Concentration, and Endotoxin Level Determination

The RPC pool was buffer exchanged to 1×DPBS (Corning Inc., New York, NY, USA; endotoxin tested) on two HiPrep 26/10 Desalting columns (GE Healthcare, Uppsala, Sweden) connected in series. The buffer exchange was performed by three consecutive injections of the RPC pool by using an ÄKTAexplore 100 system with a flow rate of 5–8 mL/min. Eluted protein from each injection was collected and pooled.

The buffer-exchanged pool of 3A3 and 3A was concentrated to 4.0 mg/mL and 2.6 mg/mL, respectively, by using an Amicon Ultra-15 centrifugal filter with 3kDa NMWL (Merck Millipore, Burlington, MA, USA). The concentration of 33A was diluted to 0.766 mg/mL in PBS containing 50 mM L-arginine to prevent aggregation. The endotoxin levels were determined to be <0.2 EU/mL for 3A3 and 3A and <0.5 EU/mL for 33A using an Endoafe PTS unit (Charles River, MA, USA) with a 1–0.01 EU/mL cartridge (Charles River, MA, USA).

### 2.5. Purity and Mass Identity Determination

The purity was determined with reverse-phase high performance liquid chromatography (RP-HPLC) on a 1200 series HPLC system (Agilent Technologies, Santa Clara, CA, USA) using an analytical Zorbax 300SB-C18 column (Agilent Technologies, Santa Clara, CA, USA) with a 20–50% acetonitrile elution gradient over 20 min with a flow rate of 1 mL/min. Electrospray ionization mass spectrometry (ESI-MS) with a 6520 Accurate-Mass Q-TOF LC/MS (Agilent Technologies, Santa Clara, CA, USA) was used to confirm the mass identity of the purified constructs.

### 2.6. Secondary Structure and Thermal Stability Analysis

Alpha-helical content, thermal stability, and refolding capacity were analyzed using circular dichroism spectroscopy on a Chirascan spectropolarimeter (Applied Photophysics, Surrey, UK) with an optical path length of 1 mm and a protein concentration of 0.25 mg/mL. The evaluation of thermal stability was performed using variable temperature measurement (VTM). The change in ellipticity at 221 nm was monitored while heating (5 °C/min) the sample from 20 to 90 °C. The approximations of the melting temperatures were made from non-linear regressions of the generated curves using a Boltzmann Sigmoidal model (GraphPad Prism, version 7, GraphPad Software, La Jolla, CA, USA). Spectra obtained from measurements at wavelengths in the range 195–260 nm at 20 °C before and after VTM were compared to assess the refoldability of the affibody molecules after thermal denaturation.

### 2.7. Affinity Determination

The binding affinity of the constructs to human HER3 (Sino Biological, Wayne, PA, USA) and murine mErbB3 (Sino Biological, Wayne, PA, USA) was evaluated with surface plasmon resonance (SPR) on a BIAcore T200 system (GE Healthcare, Uppsala, Sweden) using a CM5 sensor chip immobilized with HSA. The constructs were captured on the HSA surface, followed by multi-cycle injections of five HER3 or mErbB3 concentrations (3.125, 6.25, 12.5, 25, and 50 nM). In addition, the binding affinity to HSA and mouse serum albumin (MSA) was evaluated using the same sensor chip and a multi-cycle setup with three concentrations (3.125, 6.25 and 12.5 nM) of the constructs. All experiments were performed in duplicate. The acquired sensorgrams were analyzed using a Langmuir 1:1 kinetic model.

### 2.8. Inhibition of In Vitro Phosphorylation

The inhibition of HER3 phosphorylation on BxPC-3 cells following treatment with affibody molecules was evaluated using a Phospho-HER3 ELISA assay (R&D Systems, Minneapolis, MN, USA) according to the manufacturer’s protocol. Western blot was used to evaluate the phosphorylation status of the downstream signaling molecules AKT and ERK1/2 on the same treated samples. The pAKT antibody (15306974; rabbit monoclonal, 1/500 dilution), pERK1/2 antibody (11372362; rabbit monoclonal, 1/1000 dilution), ß-actin antibody (15284898; rabbit monoclonal, 1/2000 dilution), and anti-rabbit HRP-conjugated antibody (11859140, goat polyclonal, 1/2000 dilution) were purchased from Fisher Scientific (Hampton, NH, USA).

Treated cell samples were prepared as previously described [24]. Briefly, BxPC-3 cells were expanded in RPMI-1640 medium (Thermo Fisher Scientific, Waltham, MA, USA) supplemented with 10% FBS (Thermo Fisher Scientific, Waltham, MA, USA) and 1% penicillin–streptomycin (Thermo Fisher Scientific, Waltham, MA, USA). Cells were seeded at 10^6^ cells per well in six-well plates and allowed to adhere overnight. The medium was subsequently changed to a starvation medium (RPMI-1640 supplemented with 0.5% FBS) and incubated overnight at 37 °C. Cells were treated with seven different concentrations of either affibody construct (3A3, 33A and 3A) or MM-121 for 10 min at 37 °C. The volume in wells with untreated cells were equalized using the starvation medium. Cells were then treated with 4 nM heregulin and incubated for an additional 10 min at 37 °C. The plates were transferred to ice, lysed using Cell Lysis Buffer (Cell Signaling Technology, Danvers, MA, USA), containing 100 mM orthovanadate (BioNordika, Stockholm, Sweden), 100 mM PMSF (Sigma-Aldrich, St. Louis, MO, USA), and PhosSTOP (Sigma-Aldrich, St. Louis, MO, USA), and collected.

### 2.9. Inhibition of In Vitro Proliferation

The HER3-expressing cancer cell line BxPC-3 was cultured in RPMI-1640 medium supplemented with 10% FBS and 1% penicillin–streptomycin. The cells were treated with TrypLE Express Enzyme (Thermo Fisher Scientific, MA, USA), diluted using RPMI-1640 supplemented with 4% FBS, and seeded in 96-well plates at a density of 2000 cells per well and allowed to adhere overnight. On the subsequent day, cells were treated with ten concentrations of the affibody constructs and MM-121 (the production and characterization of the antibody is as previously described [21]) in the presence or absence of 4 nM heregulin and incubated at 37 °C for 5 days. Cell viability was determined by measuring fluorescence after incubating with alamarBlue Cell Viability Reagent (Invitrogen, Waltham, MA, USA) for 2 h.

### 2.10. Therapy Study

Female Balb/c nu/nu mice were implanted with a 100 µL suspension of 5 × 10^6^ BxPC-3 cells in PBS, and one week later (day 0) mice were randomly sorted into five groups (*n* = 9–10 per group). Tumor volume was 45 ± 20 mm^3^ and mouse weight was 16 ± 1 g at the start of the experiments. Mice were i.p. injected with 150 μL conjugate solution in PBS containing 400 µg of 3A, 600 μg of 33A, 600 μg of 3A3, or 600 µg MM-121 three times per week. The control group was injected with PBS only. Tumor dimensions were measured using digital calipers and mice status was monitored twice per week. Mice were euthanized at a predetermined humane end point (tumor volume exceeding 1 cm^3^ or ulcerated, or when the animal’s weight was reduced by 10% within one week). The practical end point was 93 days after treatment started, with the last treatment being performed on day 90. HER3 occupancy was investigated using [^68^Ga]Ga-(HE)_3_-Z_08698_-NODAGA when tumors reached 700–800 mm^3^, as described below.

At the humane end point, samples from blood serum, kidney, liver, and tumor were collected for pathological examination. Blood serum was analyzed for the concentration of urea, creatinine, aspartate aminotransferase (AST), and alkaline phosphatase (ALP) at the Department of Pathology and Wildlife Diseases, National Veterinary Institute, Uppsala, Sweden. Histological examination was performed at the same department. Hemotoxylin, eosin (HE), and HER3 immunohistochemical (IHC) staining and slide scanning were performed at the Swedish SciLifeLab facilities, as previously described [21].

### 2.11. Tumor Imaging

The labeling of (HE)_3_-Z_08698_-NODAGA with gallium-68 and micro positron emission tomography (microPET)/computed tomography (CT) imaging of HER3 expression in xenografted mice were done according to a published protocol [27]. Briefly, whole body PET scans of the BxPC-3 xenografted mice were performed under general anesthesia in a nanoScan PET/MRI system (Mediso Medical Imaging Systems Ltd., Budapest, Hungary) 1 h post i.v. injection of 2 µg of the anti-HER3 affibody imaging probe [^68^Ga]Ga-(HE)_3_-Z_08698_-NODAGA (1.6–7.3 MBq). CT acquisitions were performed using a nanoScan SPECT/CT system (Mediso Medical Imaging Systems Ltd., Budapest, Hungary) immediately after PET acquisition using the same bed position. PET scans were performed for 30 min. PET data were reconstructed into a static image using a Tera-Tomo™ 3D reconstruction engine. CT data were reconstructed using filtered back projection. PET and CT files were fused and analyzed using Nucline 2.03 Software. Imaging was performed one day after therapeutic injection.

## 3. Results

### 3.1. Characterization of Constructs

The molecular mass of each construct was determined with ESI-MS (Figure S1) and was in perfect agreement with the theoretical values (Table 1). The purity of the constructs was determined by RP-HPLC and exceeded 95% for all constructs (Figure S2). The alpha-helical content, thermal stability, and refolding capacity of the constructs were investigated with circular dichroism spectroscopy. The thermal denaturation curves are shown in Figure S3 and the associated melting temperatures are presented in Table 1. The calculated melting temperatures represent an average for the constructs as a whole because of overlapping transitions in unfolding for each individual domain. All constructs exhibited complete refolding following thermal denaturation, as is evident from spectra comparisons at 20 °C before and after denaturation, with the exception of a small shift in helicity for 3A3.

### 3.2. Affinity Determination

Kinetic data acquired from SPR analysis are presented in Table 1 as the average of duplicate injections. K_D_ values refer to the monovalent affinity for human HER3, according to a Langmuir 1:1 model. The constructs demonstrated high monovalent affinity for soluble HER3 in the low to sub-nanomolar range, while simultaneously bound to HSA, which is consistent with previous results [24]. The affinity for mErbB3 was about half that for HER3 in the case of 33A and 3A, and about four times lower in the case of 3A3, which was reflected in a more rapid dissociation. Representative sensorgrams with fitted curves for each construct are shown in Figure S4. Additionally, the affinity for both HSA and MSA was determined using SPR analysis to verify that fusion to affibody molecules does not dramatically affect the binding of the ABD. All constructs demonstrated a high picomolar affinity for HSA and sub-nanomolar affinity for MSA. The data are presented in Table 1 and sensorgrams with fitted curves are shown in Figure S5.

### 3.3. Inhibition of Phosphorylation and Proliferation In Vitro

**Figure 1:**
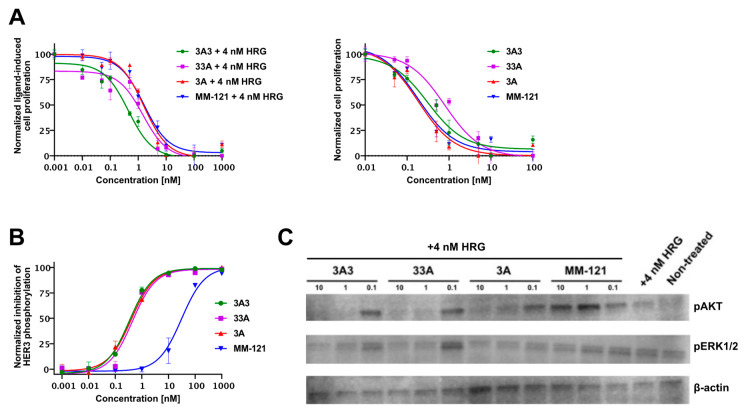
(**A**) The inhibition of cell proliferation in vitro using BxPC-3 pancreatic cancer cells by anti-HER3 affibody molecules in comparison with MM-121 in both the presence (left) and absence (right) of 4 nM heregulin. Data are presented as a dose–inhibition curve normalized to a positive heregulin-treated control. (**B**) Results from phospho-HER3 ELISA demonstrating the dose-dependent inhibition of heregulin-induced phosphorylation of HER3 following the treatment of affibody constructs and MM-121 in BxPC-3 cells. (**C**) Western blot showing downregulation of the downstream signaling molecules pAKT and pERK1/2 in BxPC-3 cells following treatment with affibody constructs and MM-121 in the presence of 4 nM heregulin. Non-treated cells were not stimulated by the addition of heregulin. ß-actin levels are included for normalization. Please note that due to the overlapping molecular weights between pERK1/2 and ß-actin, the antibody against ß-actin was not used on the membrane for pERK1/2. The loaded sample in the pERK1/2 western blot is, however, from the same lysate that was used in the western blot of pAkt and ß-actin. The intensity of ß-actin is thus used as a control for the normalization of the cell and protein amount that is also in the western blot of pERK1/2.

The inhibitory effects of the anti-HER3 affibody molecules on cell proliferation are shown in Figure 1A as a dose–inhibition curve in the presence (left) and absence (right) of 4 nM heregulin. The data are normalized to the lowest and highest values for each protein. Absolute inhibition compared to a positive heregulin-treated control is presented in Figure S6A. The experiment was performed twice in order to validate the results. The results shown in Figure S6B include fewer concentrations but demonstrate similar trends in the inhibition of cell proliferation. The effects of ligand-inhibition were more pronounced when the cells were stimulated with 4 nM heregulin compared to cells relying on the autocrine secretion of heregulin to stimulate HER3 activation. The affibody molecules and MM-121 were comparable in terms of potency. The inhibitory effects observed on cell proliferation were reflected by a dose-dependent downregulation of phosphorylated HER3, as well as active forms of signaling molecules acting downstream of the receptor in the presence of an excess of the ligand heregulin. The status of phosphorylated HER3 following treatment was determined using ELISA and the results are shown in Figure 1B. The affibody constructs demonstrated a comparable and high potency with IC50 values below 0.5 nM, whereas MM-121 demonstrated a considerably lower potency. These results were corroborated by a concomitant downregulation of downstream signaling molecules. Results from immunoblotting show a dose-dependent decrease in phosphorylated pAKT and pERK1/2 for cells treated with affibody molecules with low to undetectable levels in the 1–10 nM range. The antibody failed to demonstrate any effect at the given concentrations but did completely inhibit pAKT at a concentration of 100 nM (data not shown), which is in accordance with the lower potency for the inhibition of phosphorylated HER3.

### 3.4. Tumor Growth Inhibition in BxPC-3 Xenografted Mice

**Figure 2:**
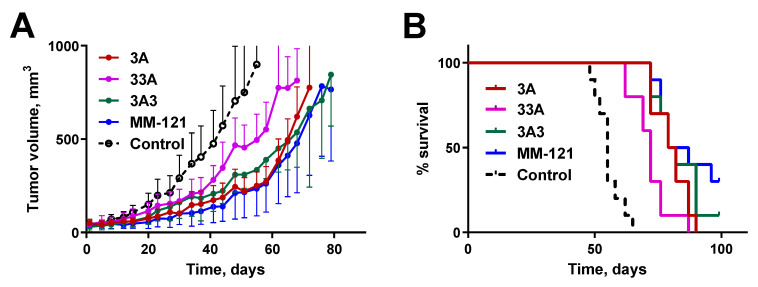
(**A**) Tumor growth inhibition in BxPC-3 xenografted mice treated with HER3-targeting affibody constructs 3A, 33A, and 3A3. Inhibition efficacy was compared with the anti-HER3 antibody MM-121. Treatment started on day 0 (7 days following the implantation of the xenograft) and mice were treated three times per week with i.p. injections (*n* = 9–10). The final treatment was performed on day 90 and the remaining mice were euthanized on day 93. Curves were interrupted when three mice in a group were euthanized (30−33%). (**B**) Survival of BxPC-3 xenografted mice (*n* = 9–10) treated with HER3-targeting affibody constructs 3A (average survival 80.5 days, significantly longer than 33A and control), 33A (72 days, significantly longer than control), 3A3 (80.5 days, significantly longer than 33A and control), and MM-121 (83 days, significantly longer than 33A and control). The control group had an average survival of 55 days.

In vivo efficacy of the tumor growth inhibition of the new HER3-targeting affibody constructs 3A and 33A was compared with the previously evaluated construct 3A3 and the anti-HER3 antibody MM-121 in mice bearing HER3-expressing BxPC-3 xenografts (Figure 2A). A cytostatic effect of the therapeutic constructs was observed as early as 2–3 weeks after the onset of therapy (Figure 2A). The effect from treatment with 3A and 3A3 was more pronounced than with 33A and equal to the effect from the anti-HER3 antibody MM-121. Average tumor volumes were significantly smaller in groups treated with 3A, 3A3, and MM-121 than in control group on day 15, and in the group treated with 33A on day 30. With time, this tendency became stronger, and on day 58, the average tumor volumes in the group treated with 33A were significantly bigger than in the groups treated with 3A and MM-121.

Visual control, the monitoring of the individual animal weight curves (Figure S7), the biochemical examination of blood serum, and the pathological examination of kidneys and livers showed no difference between treated and control animals. Blood concentrations of urea, creatinine, alkaline phosphatase (ALP) and alanine aminotransferase (ALAT) in treated animals were comparable with levels found in animals in the control group (data not shown). The liver and kidneys did not show significant histological lesions. The parenchymal cells in both organs remained normal and there was no evidence of toxic damage.

**Figure 3:**
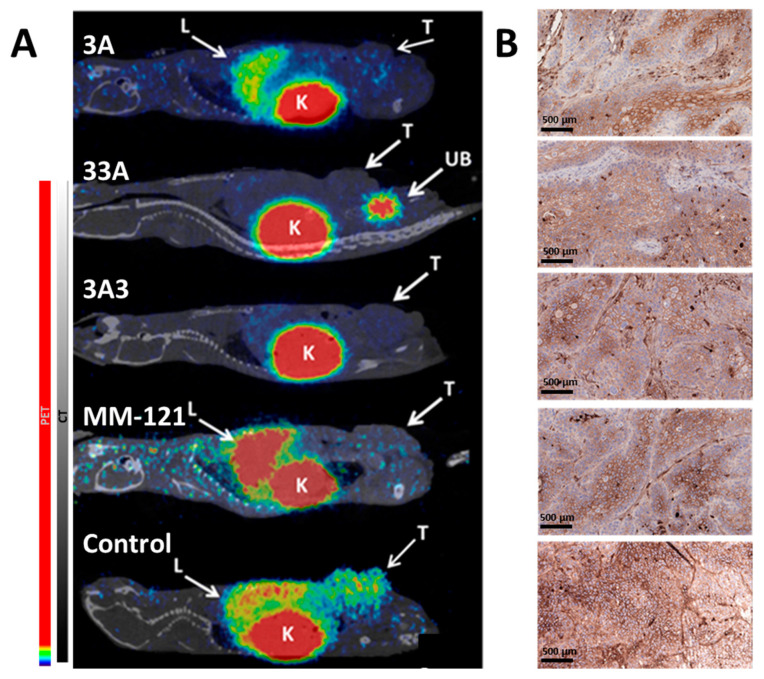
(**A**) The micro positron emission tomography (microPET)/computed tomography (CT) images (MIP) of the BxPC-3 xenografted mice 1 h post i.v. injection of 2 µg anti-HER3 affibody imaging probe [^68^Ga]Ga-(HE)_3_-Z_08698_-NODAGA. Images were performed 1 day after therapeutic injection. Tumors (T), livers (L), kidneys (K), and urinary bladders (UB) are indicated. (**B**) Immunohistochemical staining of HER3 expression in BxPC-3 xenografts. Samples were taken at end point.

When tumors reached 700 cm^3^ (about 4 days before humane end point) the HER3 expression in tumors was visualized using a HER3-targeting affibody molecule [^68^Ga]Ga-(HE)_3_-Z_08698_-NODAGA in two mice per group (Figure 3A). HER3 expression in xenografts was clearly seen in control animals but not in treated groups. Among healthy organs, activity uptake was detected in kidneys in all groups, but hepatic uptake could only be visualized in the control group and groups treated with MM-121 and 3A. Activity uptake was lower in the latter group.

All treated groups had significantly longer (***) median survival times than the control group (Figure 2B). Among the treated groups, a significantly shorter (*) median survival time was found for the bivalent 33A construct. All animals were euthanized when tumor volumes reached the critical volume (1000 mm^3^), except for two mice in the control group that had internal bleeding due to extensive metastatic spread. One mouse in the group treated with 3A3 and three mice in the group treated with MM-121 had tumors that did not reach critical mass at the end point of the experiment (Figure S8).

Cytopathological examination of tumors dissected post mortem showed that tumors displayed features consistent with carcinoma, and nuclear and cytoplasmic features were consistent with highly malignant cells. Some tumors showed cystic structures of varying size lined by cubic, cylindric, or flattened epithelial cells. These structures contained mucous or proteinaceous secretions, or blood mixed with damaged or necrotic/apoptotic cells. These cystic structures were more common in the control group. Squamous metaplasia with or without keratin production was also observed. Mitotic activity was high or very high in all groups. Cells grew in lobules of varying sizes enclosed by thin septa of connective tissues, but some tumors in the treated groups exhibited a well-developed stroma with rich amounts of collagen fibers. Tumors in the groups treated with 3A3 and MM-121 had more stroma than tumors in the groups treated with 3A and 33A. Squamous metaplasia with or without keratin production was also observed, especially in the group treated with MM-121. Tumors showed apoptotic cells, areas of necrosis, and inflammatory cell infiltrates that were more common in the groups treated with 3A, 3A3, and MM-121. The inflammatory cell reaction was most prevalent at the tumor periphery. However, there were no remarkable histological differences between the different experimental groups.

The immunohistochemical examination of excised tumors showed different patterns of HER3 expression in control and treated xenografts (Figure 3B). While xenografts in the control group exhibited homogeneous HER3 expression, the expression in treated xenografts was weaker in the periphery of lobules. No significant differences were observed in the mErbB3 expression in livers between control and treated mice (data not shown).

## 4. Discussion

The overexpression of HER3 is a potential cause for therapy resistance and a driver for tumor progression in many cancers [12]. HER3 has thus attracted attention as a molecular target for cancer therapy. While several HER3-targeting therapeutic agents are currently under preclinical and clinical development, no HER3-specific drugs or treatments have yet been approved [12]. Efforts by our group and others have demonstrated in preclinical models that anti-HER3 affibody molecules could be an alternative for HER3-targeted therapy [21,27,28]. For example, the affibody construct TAM-HER3 (an ABD flanked by two HER3-binding affibody molecules) delayed the growth of HER3 expressing tumors in a mouse model with equal potency as the HER3-targeting antibody MM-121 [21], indicating the main mode of action is based on receptor blocking and is not Fc-mediated. It should also be noted that MM-121 (seribantumab) was in an IgG2 format for the clinical evaluations in order to minimize potential Fc-mediated adverse effects, indicating that the biological effect from MM-121 is largely driven by ligand blocking [29]. In an effort to optimize the format of TAM-HER3, we hypothesized that the arrangement of the molecular building blocks could affect the therapeutic efficacy of the construct. The comparison of five different ABD-fused monovalent and bivalent anti-HER3 affibody constructs [24] demonstrated that tumor uptake, clearance, and tumor retention were significantly influenced by valency and the position of the ABD.

In addition to the previously investigated TAM-HER3 (3A3), variants with an ABD fused to the C-terminus (3A, 33A) showed promising biodistribution profiles and inhibition of heregulin-induced phosphorylation in HER3-expressing BxPC-3 cells [24]. Based on this, we decided to investigate the therapeutic efficacy of these HER3-targeting affibody constructs in vivo using a preclinical pancreatic cancer model, together with the therapeutic HER3-targeting monoclonal antibody MM-121 as a comparator.

Treatment with all affibody variants was well tolerated, as corroborated by animal weight curves, the analysis of blood serum, and liver and kidney pathology. Tumor growth data for MM-121 and the 3A3 construct were comparable to previously reported results [21]. All three affibody variants demonstrated a therapeutic effect, e.g., significantly delayed tumor growth and prolonged survival compared to the control (PBS). This was in agreement with the results from in vitro experiments showing the dose-dependent inhibition of cell proliferation and HER3 phosphorylation. Furthermore, western blot analysis showed a downregulation of the downstream signaling molecules pAKT and pERK1/2, which was in good agreement with the downregulation of pHER3 observed from ELISA at the given concentrations.

While there was no significant difference between the affibody variants in vitro, the molecular design of the affibody molecules influenced the anti-proliferative efficacy and median survival in vivo. Among the tested variants, 33A was the least potent in terms of tumor growth inhibition and prolonging survival. Tumors in animals treated with 33A were significantly smaller (volume in mm^3^) than in the control only 30 days after the start of treatment, compared to 14 days for 3A, 3A3, and MM-121. Furthermore, after 58 days, there was a significant separation in tumor volume curves between 33A- and 3A- or MM-121-treated groups. Animals treated with 33A also had a significantly shorter median survival than animals treated with 3A, 3A3, and MM-121. The lower in vivo efficacy of 33A could be explained by a more rapid blood clearance, and thus reduced bioavailability compared to 3A and 3A3, which was previously reported by our group [24].

Comparing the tested affibody variants with the HER3-targeting affibody MM-121, we could conclude that treatment with the monovalent 3A variant was equally potent as the treatment with MM-121, both regarding absolute tumor volume and median survival. This could partially be attributed to the longer circulation of 3A in the blood [24] and a higher monovalent affinity for HER3 compared to 3A3 and 33A. The slower clearance potentially provides a more constant supply to the tumor and the increased affinity could prolong its retention in tumors. A longer residence time in blood might also explain why MM-121 demonstrated a high in vivo efficacy, despite significantly lower IC50 values in the phospho-ELISA assay.

Multivalence did not appear to improve the therapeutic efficacy of our affibody molecules. This is particularly interesting, because our findings contradict data published by Schardt et al. [27,28], who investigated the therapeutic efficacy of multivalent anti-HER3 affibody constructs using the HER3-binding variant Z_05413_. The authors hypothesized that dimerization would improve affinity through avidity and potentially increase the inhibition of HER3 phosphorylation, downstream signaling, and tumor progression and therefore improve the therapeutic efficacy [27,28]. Indeed, the authors demonstrated a significant reduction in tumor progression compared to controls for groups treated with bivalent affibody constructs but not with monovalent constructs [27]. It should be noted that in the study, in vitro efficacy was dependent on cell lines being autocrine for heregulin [28] and in vivo experiments were performed using an ovarian cancer model with OvCAR8-derived ADR-RES xenografts [27]. The different in vivo model could be a possible explanation for the deviating results, as the density of HER3 receptors could affect the ability of bivalent variants to bind two HER3 receptors simultaneously. In the present study, the lower HER3 receptor density on BxPC-3 cells might prevent the bivalent binding of the 33A and 3A3 constructs, in which case the smaller size of 3A could be beneficial for efficient tumor penetration and extravasation.

The observation of more prevalent cystic structures in tumors in the control group and more apoptotic cells and inflammations in the treated groups suggest that the evaluated constructs have a therapeutic effect on cell growth and survival, which is in agreement with the in vitro data demonstrating the inhibition of HER3 phosphorylation and of downstream signaling molecules. In the histopathological examination of tumor material, it was found that treatment with the affibody constructs and MM-121 cause the downregulation of HER3, together with inflammatory cell reactions in the periphery of treated tumors (but not in the center of tumor cell islands), indicating a limited penetration depth of the molecules. The downregulation of HER3 expression in response to HER3-targeted therapy has previously been observed [21,27,30]. In contrast to our findings however, Schardt et al. [27,28] reported HER3 downregulation mainly by the multimeric HER3-targeting affibody constructs, but no or little effect was observed for the monomeric affibody. It was hypothesized that multivalence is required to trigger the sequestration of HER3 receptors, but from our data it could be speculated that the multivalence of the therapeutic agent is not essential for the downregulation of HER3 and for achieving a therapeutic effect. The larger size of the multimeric affibody constructs (3A3, 33A) could possibly negate the positive effects of potential avidity by reducing tissue penetration and extravasation compared to the monovalent 3A. Smaller size is one of the potential advantages of using smaller scaffold proteins rather than antibodies.

In addition to the evaluation of the therapeutic efficacy of the affibody constructs, we also demonstrated the feasibility of the radiolabeled anti-HER3 conjugate ^68^Ga-(HE)_3_-Z_HER3_-NODAGA as a companion diagnostic agent of HER3 expression and of the monitoring of receptor occupancy. ^68^Ga-(HE)_3_-Z_HER3_-NODAGA was developed and preclinically evaluated for the imaging of HER3 expression [31]. As expected, the control animal showed activity uptake in the non-treated HER3 expressing xenografts, as well as the liver and kidneys. The renal pathway is the main elimination pathway for affibody molecules and the liver naturally expresses mErbB3 (murine ortholog of HER3), resulting in tracer accumulation in these organs. No activity uptake of ^68^Ga-(HE)_3_-Z_HER3_-NODAGA was observed in the tumors of animals in the treated groups, demonstrating the occupation of HER3 receptors by the therapeutic agents. PET imaging showed no differences between the bivalent affibody constructs, but revealed hepatic uptake of ^68^Ga-(HE)_3_-Z_HER3_-NODAGA in animals treated with MM-121 and 3A. MM-121 is not cross-reactive to mErbB3, which explains the activity accumulation in the liver. The hepatic uptake was lower in animals treated with 3A than in control animals or animals treated with MM-121. This low, but not negligible, hepatic uptake could be explained by differences in cellular processing in tumor and normal cells after the binding of anti-HER3 affibody molecules. Despite similar values of binding affinities both to mErbB3 and MSA for all tested affibody constructs (Table 1), their blood clearance rate and hepatic uptake differed dramatically [24]. The monovalent variant 3A had significantly higher blood concentration and significantly lower hepatic uptake 24 h p.i. than the bivalent variants 33A and 3A3 [24]. It has previously been shown that a trimeric anti-HER3 affibody (without an ABD) had a 10-fold higher hepatic uptake than a monomeric affibody, supposedly due to the slower dissociation of the multivalent affibody and HER3 sequestration [32]. It could be speculated that the bivalent therapeutic variants have a similar effect, and as a result, the hepatic uptake of ^68^Ga-(HE)_3_-Z_HER3_-NODAGA was observed in the 3A treated group, but not the 3A3 and 33A groups. Additionally, the images confirmed that the affibody constructs and MM-121 bind to the same epitope of the HER3 receptor, as was shown previously in vitro [33]. This demonstrates that anti-HER3 affibody-based imaging agents could be used as highly sensitive and specific diagnostics for both patient stratification and therapy monitoring in therapy studies involving MM-121.

These results extend the work conducted by Schardt et al. evaluating the efficacy of mono- and bivalent HER3-targeting affibody constructs in an ovarian cancer xenograft model as monotherapies and in combination with carboplatin [27]. This study demonstrates the plausibility of affibody molecules as HER3-targeting therapeutic agents for the inhibition of HER3-mediated tumor growth and corroborates previously obtained in vivo efficacy data pertaining to the structurally similar TAM-HER3 [21].

## 5. Conclusions

In conclusion, we have evaluated the influence of the molecular design of HER3-targeting mono- and bivalent affibody constructs on therapeutic efficacy in a preclinical model. While all tested variants delayed the growth of HER3-expressing xenografts, the molecular design affected the treatment efficacy in vivo, accentuating the importance of molecular design and the discrepancies that can arise when translated into an in vivo therapy setting. Treatment efficacy might be dependent on the tumor model, mono- or multivalence, the residence time of the therapeutic construct in circulation, and affinity. The monovalent Z_HER3_-ABD conjugate matched the therapeutic efficacy of MM-121 in vivo. Overall, the results of this study further demonstrate the potential of HER3-targeting affibody constructs for HER3-targeted therapy.

## Figures and Tables

**Table 1:** Biophysical characteristics. The theoretical molecular weight (Mw), melting temperature (T_m_), and K_D_ values for HER3, mErbB3, human serum albumin (HSA) and mouse serum albumin (MSA).

Constructs	Mw (Da)	T_m_ (°C)	K_D_ HER3 (nM, mean ± SD)	K_D_ mErbB3 (nM, mean ± SD)	K_D_ HAS (nM, mean ± SD)	K_D_ MSA (nM, mean ± SD)
3A3	18,971.52	57.54	1.73 ± 0.77	7.93 ± 0.95	0.011 ± 0.003	0.54 ± 0.008
33A	18,971.52	64.92	1.04 ± 0.3	2.47 ± 0.25	0.006 ± 0.002	0.35 ± 0.002
3A	12,040.78	59.32	0.51 ± 0.08	1.01 ± 0.15	0.014 ± 0.002	0.16 ± 0.001

## Supplementary Materials

The following are available online at https://www.mdpi.com/1999-4923/12/6/551/s1, Figure S1. Mass identity determination. The mass of each construct was determined using ESI-MS, which was in perfect agreement with theoretical values (Table 1). Figure S2. Purity analysis of the HER3-targeting ABD-fused affibody molecules was performed using analytical HPLC using absorbance measurement at 220 nm. Figure S3. Secondary structure and thermal stability analysis of HER3-targeting ABD-fused affibody molecules using circular dichroism. Figure S4. Representative sensorgrams with fitted curves from SPR analysis to determine affinity for HER3 and mErbB3. Figure S5. Representative sensorgrams with fitted curves from SPR analysis to determine affinity for HSA and MSA. Figure S6. (A) Data in figure 1 presented as absolute inhibition of cell proliferation normalized to positive heregulin-treated control in the presence and absence of 4 nM heregulin. (B) Duplicate experiment for inhibition of cell proliferation performed at a separate occasion with fewer concentrations, normalized to highest and lowest values for each protein. Figure S7. Average body weight as an indication of toxicity during treatment. Mice weights were determined twice weekly and values are presented as average weight in groups (n = 9−10) ± SD. Figure S8. Individual tumor growth.
