# Supplementary material for: Evaluating the Therapeutic Efficacy of Mono- and Bivalent Affibody-Based Fusion Proteins Targeting HER3 in a Pancreatic Cancer Xenograft Model
Source: Pharmaceutics. 2020 Jun 13;12(6):551. doi: 10.3390/pharmaceutics12060551 (PMC7356278; doi:10.3390/pharmaceutics12060551)
Supplement: Supplementary file 1 [file pharmaceutics-12-00551-s001.pdf]

pharmaceutics

MDPI

Supplementary Materials: Evaluating the Therapeutic Efficacy of Mono- and Bivalent Affibody-Based Fusion Proteins Targeting HER3 in a Pancreatic Cancer Xenograft Model

Charles Dahlsson Leitao, Sara S. Rinne, Mohamed Altai, Olga Vorontsova, Finn Dunås, Per Jonasson, Vladimir Tolmachev, John Löfblom, Stefan Ståhl and Anna Orlova

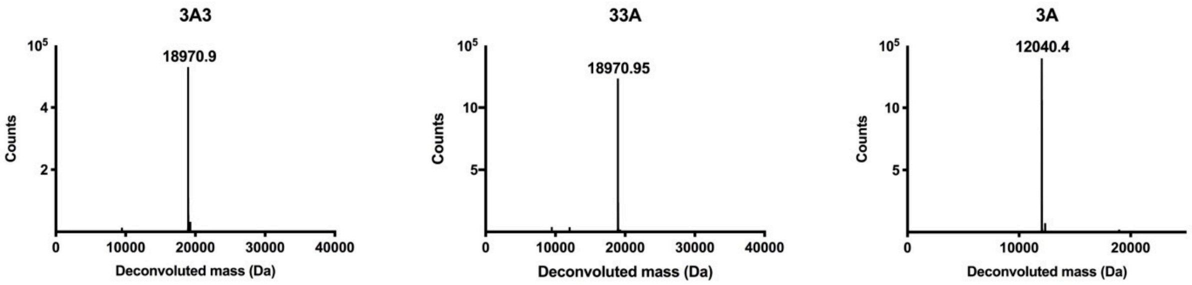

Figure S1. Mass identity determination. The mass of each construct was determined using ESI-MS, which was in perfect agreement with theoretical values (Table 1).

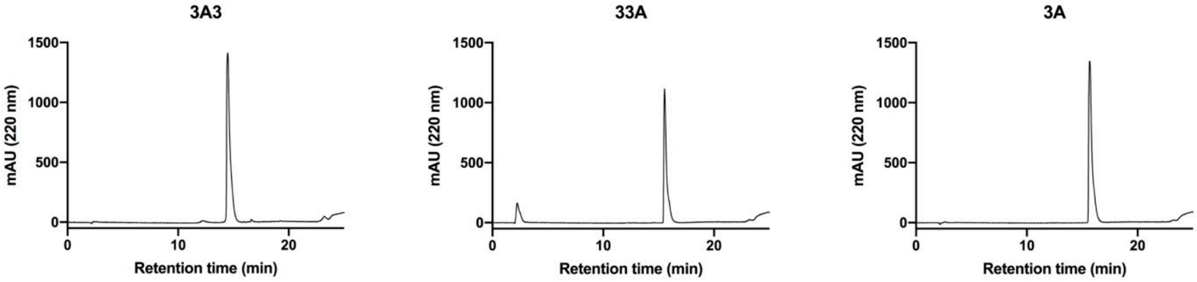

Figure S2. Purity analysis of the HER3-targeting ABD-fused affibody molecules was performed using analytical HPLC using absorbance measurement at 220 nm.

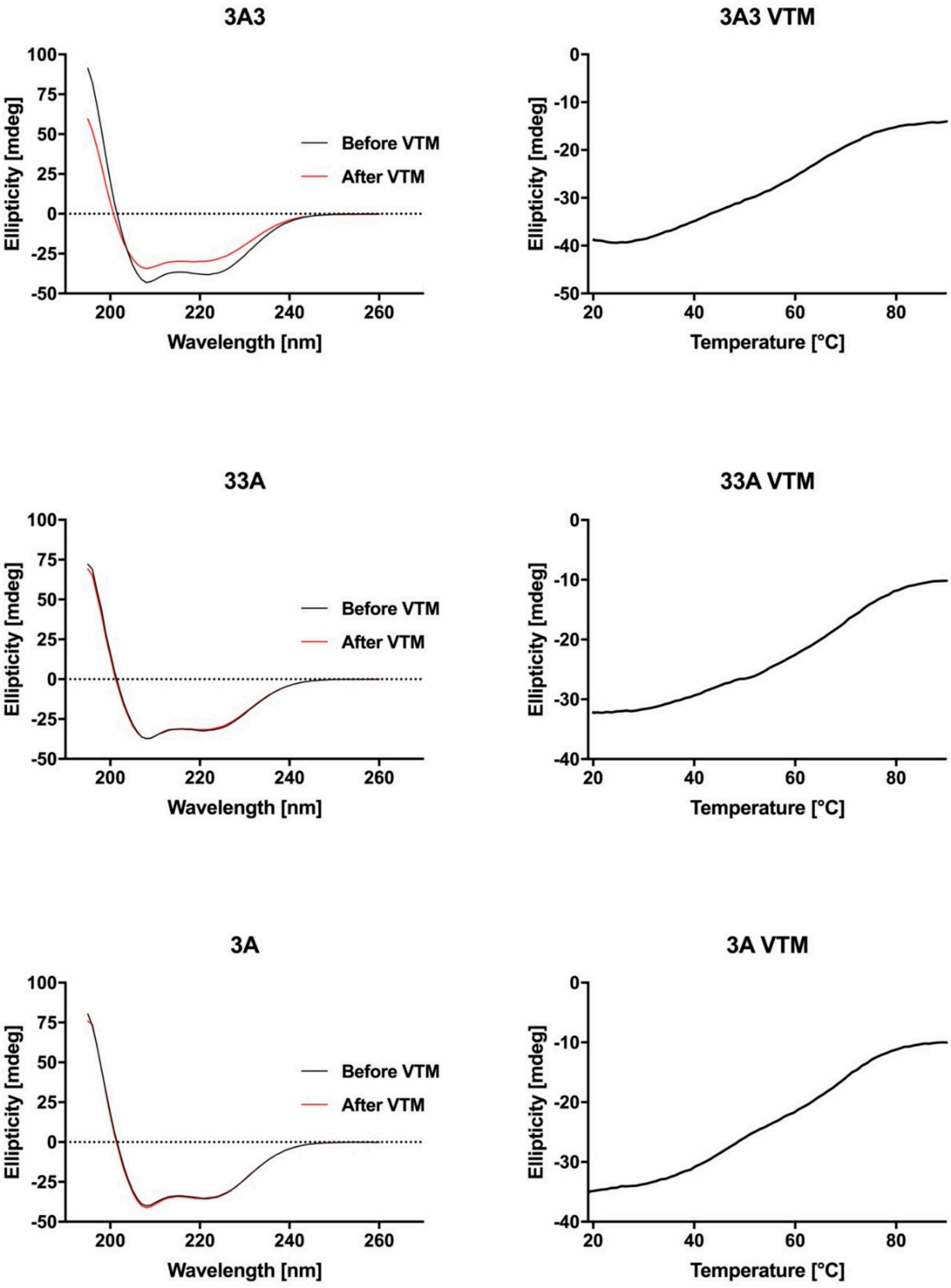

Figure S3. Secondary structure and thermal stability analysis of HER3-targeting ABD-fused affibody molecules using circular dichroism.

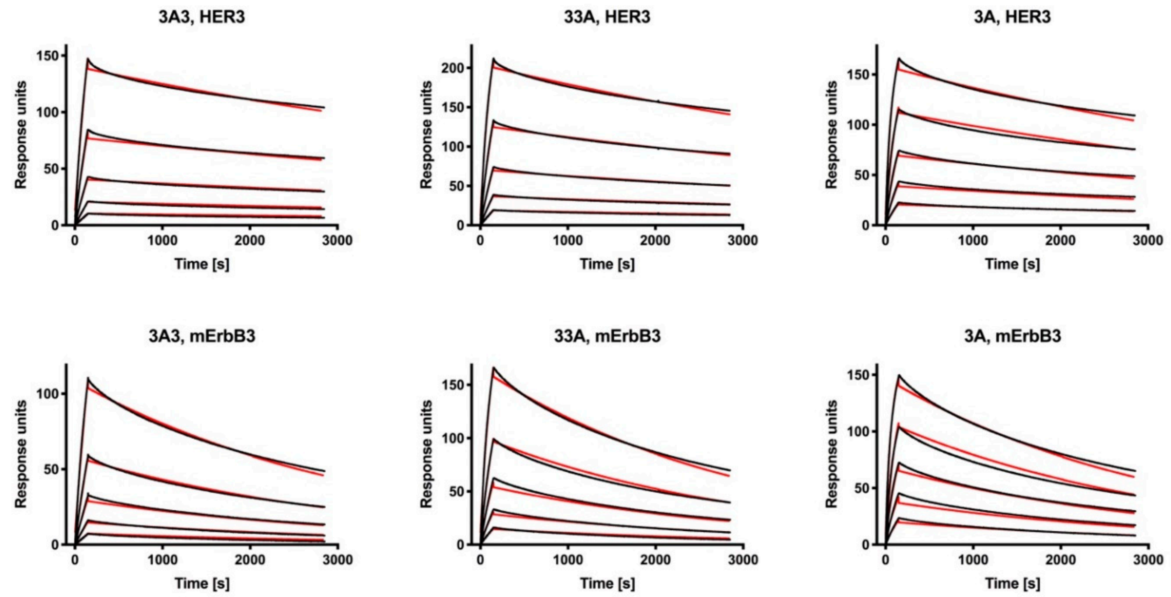

Figure S4. Representative sensorgrams with fitted curves from SPR analysis to determine affinity for HER3 and mErbB3.

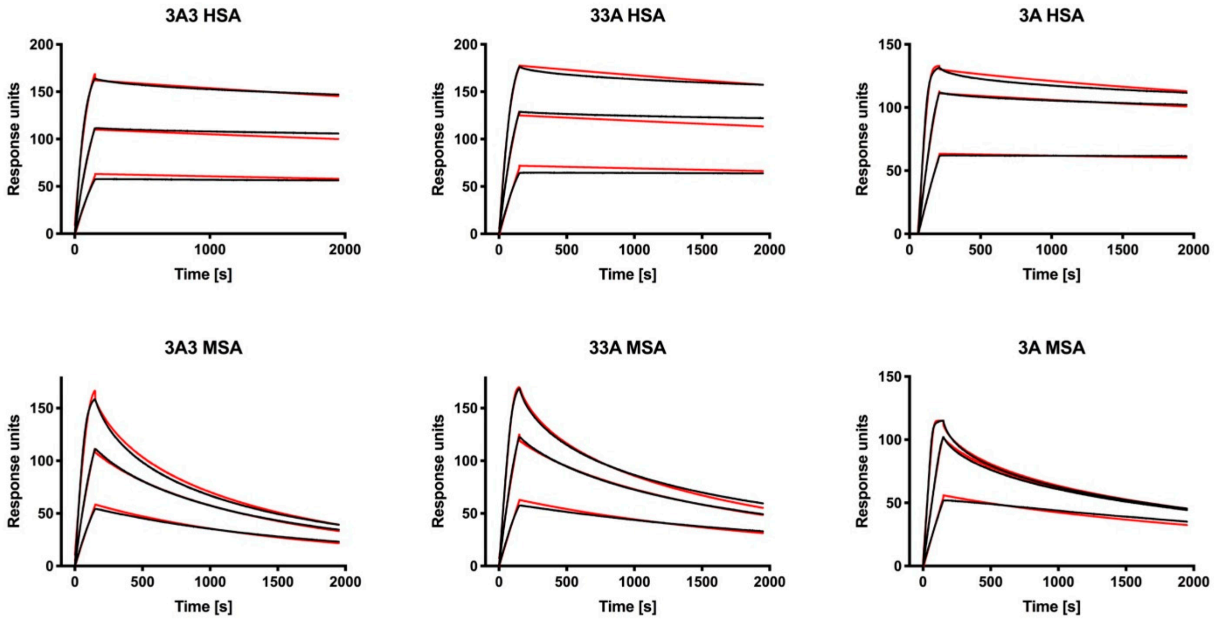

Figure S5. Representative sensorgrams with fitted curves from SPR analysis to determine affinity for HSA and MSA.

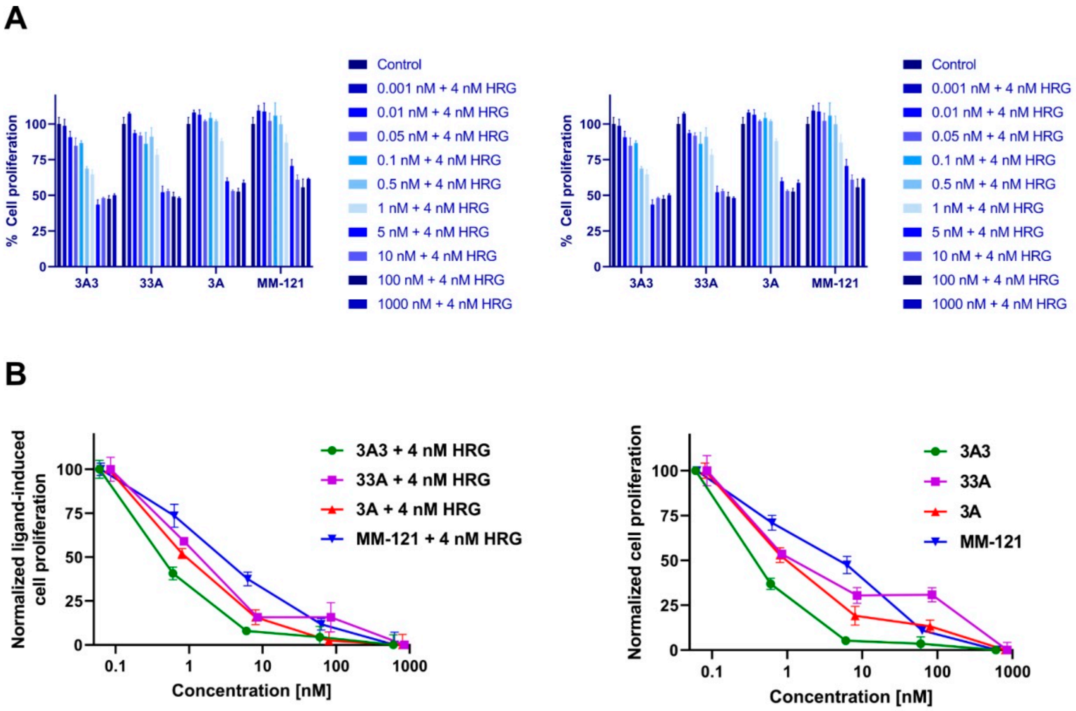

Figure S6. (A) Data in figure 1 presented as absolute inhibition of cell proliferation normalized to positive heregulin-treated control in the presence and absence of 4 nM heregulin. (B) Duplicate experiment for inhibition of cell proliferation performed at a separate occasion with fewer concentrations, normalized to highest and lowest values for each protein.

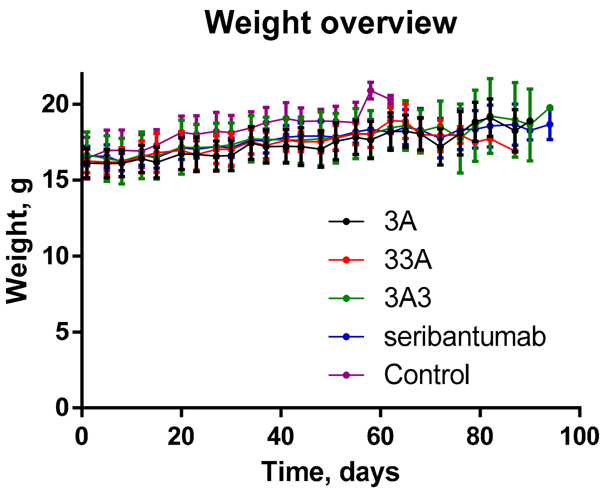

Figure S7. Average body weight as an indication of toxicity during treatment. Mice weights were determined twice weekly and values are presented as average weight in groups (n = 9–10) ± SD.

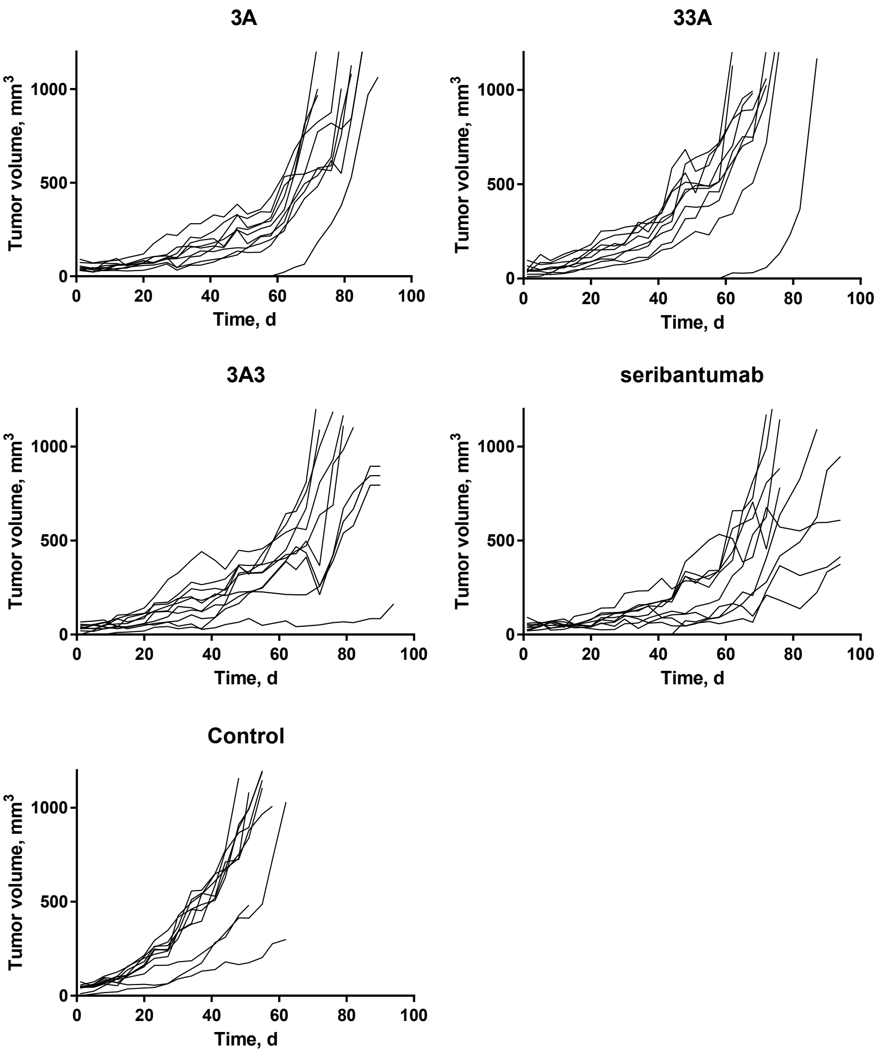

Figure S8. Individual tumor growth.

© 2020 by the authors. Submitted for possible open access publication under the terms and conditions of the Creative Commons Attribution (CC BY) license (http://creativecommons.org/licenses/by/4.0/).
